# Sickness absence and disability pension in white-collar workers in the trade and retail industry

**DOI:** 10.1093/eurpub/ckac130.123

**Published:** 2022-10-25

**Authors:** K Farrants, K Alexanderson

**Affiliations:** Division of Insurance Medicine, Department of Clinical Neuroscience, Karolinska Institutet, Stockholm, Sweden

## Abstract

**Background:**

Very little is known about sickness absence among white-collars workers in the trade and retail industry, despite being a large and important group on the labour market. The aim was to investigate future sickness absence (SA) and disability pension (DP) in a cohort of privately employed white-collar employees in the trade and retail industry.

**Methods:**

A prospective population-based cohort study of all 192,077 such white-collar workers (44% women) in Sweden in 2012, using linked microdata from three nationwide registers covering 2012-2016. Prevalence and mean number of SA and/or DP net days/year in general and by diagnoses categories were calculated for all and also stratified by sex. Logistic regression was used to calculate odds ratios (OR) and 95% confidence intervals (CI) between sociodemographic and work-related factors and future SA/DP.

**Results:**

The proportion who had SA and/or DP was higher in women (10-13%, depending on year) than men (4-6%) each year. Each studied year, women had more mean SA/DP net days than men in the entire cohort, however, among those who had SA and/or DP, there were no gender differences regarding the mean number of net days. The mean number of SA/DP net days increased for both women and men each year, especially SA due to mental diagnoses. SA in 2012 was the strongest factor associated with SA/DP in 2016 (OR women 3.28, 95% CI 3.09-3.47; men 4.10, 95% CI 3.76-4.48). Work-related factors were only weakly or insignificantly associated with future SA/DP. The ORs for most factors were stronger for men than for women.

**Conclusions:**

More knowledge about the mechanisms behind these results are needed. Some SA/DP measures showed large sex-differences, others not - it is important to use different measures to show the complexity of these phenomena. Several factors were more strongly associated with SA/DP among men than among women, indicating that there are other factors of importance for women.

**Key messages:**

- Most white-collar workers in the trade and retail industry had no SA/DP in any of the six studied years. SA due to mental diagnoses was the largest and fastest-growing SA/DP diagnostic group.
- Sickness absence in 2012, female sex, and low education were associated with future SA/DP. Most other sociodemographic and job-related factors were not, or only weakly, associated with future SA/DP.

